# Underreported and taxonomically problematic: characterization of sanguinicolid larvae from freshwater limpets (Burnupiidae), with comments on the phylogeny and intermediate hosts of sanguinicolids

**DOI:** 10.1017/S003118202300121X

**Published:** 2024-01

**Authors:** James Omondi Outa, Annemariè Avenant-Oldewage

**Affiliations:** Department of Zoology, University of Johannesburg, Auckland Park B-2006, Johannesburg, South Africa

**Keywords:** biodiversity, blood flukes, brevifurcate cercaria, *Burnupia*, Crocodile River, lophocercous-apharyngeate, rDNA sequences, scanning electron microscopy, Vaal River

## Abstract

Blood flukes of freshwater fish are understudied worldwide. Consequently, genetic information and data on their intramolluscan stages are scarce. In the current study, freshwater limpets of the genus *Burnupia* (Burnupiidae) from South Africa were examined for digeneans. Of 1645 specimens, 3.10% were infected by Sanguinicolidae larvae. Four sanguinicolids were distinguished by body size, number of penetration glands, tegumental spines’ patterns and relative sizes of the finfolds on the body and furcae. Analyses of 28S, 18S and ITS rDNA sequences showed that the morphotypes were distinct from each other and from sanguinicolids whose genetic data are available. The present study is the first genetic characterization of sanguinicolids from Africa. Phylogenetic reconstruction revealed that the present species clustered with a sanguinicolid from Poland and were sister to *Sanguinicola* and *Pseudosanguinicola* from Russia and USA, respectively. The results indicate that the current species represent an unknown genus. What is more, blood fluke sequences from East Africa (presumed to be sanguinicolids), were distant from Sanguinicolidae and showed a closer relationship with acipensericolids from the USA. Freshwater fish blood flukes seem to be more diverse than previously recorded and use species of at least 13 gastropod families as intermediate hosts.

## Graphical abstract

**Figure:**
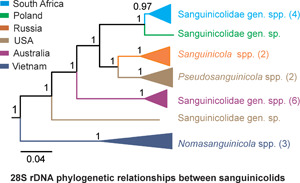

## Introduction

Fish blood flukes have 2-host life cycles in which cercariae from the intermediate hosts (gastropods, bivalves and polychaetes) penetrate the definitive hosts directly (Frandsen and Christensen, 1984; Warren and Bullard, 2019). The adult flukes inhabit the host's blood vessels, heart, liver and other highly vascularized organs (Paperna, 1996; Bullard and Overstreet, 2008). The flukes are associated with losses in aquaculture due to mortalities of the infected fish (Paperna, 1996; Bullard and Overstreet, 2008). The taxonomy of these important flukes has been problematic due to the paucity of genetic data and inadequate descriptions of putative species. Until recently, all blood flukes of fish were lumped in the family Aporocotylidae Odhner, 1912. However, 28S rDNA phylogenetic analyses of all the available sequences of marine and freshwater fish blood flukes revealed 5 distinct lineages (Cutmore *et al*., 2023). Consequently, based on morphological characteristics and genetic data, Warren and Bullard (2023) revised fish blood flukes into 5 families: Aporocotylidae Odhner, 1912; Sanguinicolidae Poche, 1926; Chimaerohemecidae Yamaguti, 1971; Acipensericolidae Warren and Bullard, 2023 and Elopicolidae Warren and Bullard, 2023.

Compared with their marine counterparts, blood flukes of freshwater fish are understudied (Zhokhov *et al*., 2021). Species of Sanguinicolidae and Acipensericolidae predominantly infect freshwater fishes (Warren and Bullard, 2023). Unlike acipensericolids that have been reported only from the USA, sanguinicolids occur in Africa, Asia, Australia, Europe, North and South America (Orélis-Ribeiro and Bullard, 2015; Zhokhov *et al*., 2021; Warren *et al*., 2023; Warren and Bullard, 2023). Despite being widespread, data on the taxonomy and ecology of sanguinicolids are scanty. According to Zhokhov *et al*. (2021), the deficiency of data might be due to their small size (1–2 mm), transparent nature and because they are easily lost through the breakage of blood vessels during parasitological examination of fish. What is more, there are little data on larval stages of fish blood flukes, and the intermediate hosts are largely unknown. This can be attributed to difficulties in identifying their larvae which often have a very low prevalence in the intermediate hosts (Bullard and Overstreet, 2008). In addition to methodological challenges that hinder the examination of sanguinicolids, there are very few parasitologists who study fish blood flukes (Warren *et al*., 2023).

Genetic data of adult sanguinicolids are available only for 7 species. They are: *Sanguinicola volgensis* (Rašín, 1929) and *Sanguinicola plehnae* Warren and Bullard, 2023 from Russia; *Nomasanguinicola canthoensis* Truong and Bullard, 2013 and 2 unidentified Sanguinicolidae spp. from Vietnam; *Pseudosanguinicola occidentalis* Warren and Bullard, 2023 and unidentified species from the USA (Warren *et al*., 2023; Warren and Bullard, 2023). In addition, DNA data are available for cercariae of 12 sanguinicolids from freshwater gastropods from Australia, East Africa, Poland and USA (Olson *et al*., 2003; Brant *et al*., 2006; Zemmer *et al*., 2020; Preston *et al*., 2021; Cutmore *et al*., 2023). However, the identities of some of the cercariae whose sequences are available remain uncertain. For instance, Orélis-Ribeiro *et al*. (2014) raised concerns about the identity of *Sanguinicola* cf. *inermis* from Poland (Olson *et al*., 2003) due to the absence of morphological data. Also, there were significant morphological variations among the cercariae from East Africa, that were described by Brant *et al*. (2006), indicating that they belong to separate and distant taxa. Herein, detailed descriptions of sanguinicolid cercariae from South African freshwater limpets are presented. The taxonomic study of the specimens was based on the integration of morphometric descriptions using light and scanning electron microscopy (SEM) and genetic characterization. Moreover, phylogenetic reconstruction has been used to explain the relationship between the present isolates and the other blood flukes of freshwater fish, for which DNA sequences are available.

Most investigations on gastropod–trematode associations in Africa have focused on selected snails belonging to the families Bulinidae, Planorbidae, Lymnaeidae and Thiaridae (Brown, 1994; Outa *et al*., 2020). Other groups of snails, particularly limpets of the family Burnupiidae, are understudied. Burnupiids are small-sized limpets whose shells rarely reach 10 mm in length (Brown, 1994). The family has 1 genus (*Burnupia* Walker, 1912), from which 22 species have been described (MolluscaBase eds., 2021). Except for *Burnupia ingae* Lanzer, 1991 from Brazil, the distribution of *Burnupia* spp. is restricted to eastern, central and southern Africa (Bouchet *et al*., 2017). Since they occur in submerged habitats and due to their small size, burnups often go unnoticed in field surveys (De Kock and Wolmarans, 2009; Outa and Avenant-Oldewage, 2023). To our knowledge, there are only 2 studies on the digeneans that are transmitted by burnupiids. Faust (1926) described a xiphidiocercaria and a brevifurcate, monostome, apharyngeate-lophocercous cercaria from *Burnupia trapezoidea* (Boettger, 1910) and *Burnupia capensis* (Walker, 1912) in South Africa. Porter (1938) reported 9 cercarial types in *Burnupia verreauxii* (Bourguignat, 1853), *Burnupia gordonensis* (Melvill and Ponsonby, 1903), *Bur*. *trapezoidea* and *Bur*. *capensis* from selected water bodies in South Africa. The digeneans reported by Faust (1926) and Porter (1938) were classified in the placeholder genus ‘*Cercaria*’ based on cercarial morphology. Consequently, the species identity, genera and families of those digeneans remain unresolved. Unfortunately, nearly 9 decades since the original studies (Faust, 1926; Porter, 1938), digeneans of African limpets have not been revisited. The current study is a report of 4 new lineages of Sanguinicolidae from *Burnupia transvaalensis* (Craven, 1881), *Burnupia mooiensis* (Walker, 1912) and *Bur*. *trapezoidea*, which are endemic in South Africa.

## Materials and methods

### Sampling and morphological analyses

**Figure 1.**
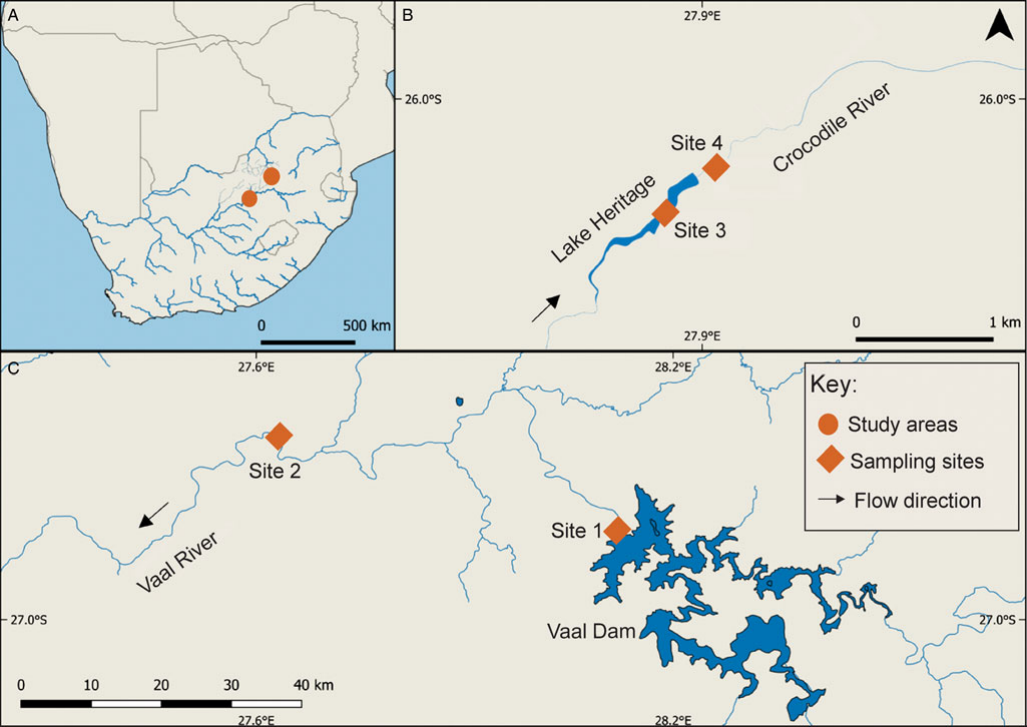
Map of southern Africa (A) and the study areas (B and C). Site 1: below the Vaal Dam (26.872364 °S, 28.117173 °E); site 2: below the Vaal River Barrage Reservoir (26.734854 °S, 27.634372 °E); site 3: Lake Heritage (25.959696 °S, 27.855555 °E) and site 4: below Lake Heritage (25.957086 °S, 27.858308 °E).

The study was conducted in 2 river systems in South Africa: Vaal River in the Orange River System and Crocodile River, which is part of the Limpopo River system. Snails were collected from 4 sites, 2 each from the Vaal and Crocodile River (Fig. 1). Site 1 is situated below the Vaal Dam wall (26.872364 °S, 28.117173 °E); site 2 is located below the Vaal River Barrage Reservoir (26.734854 °S, 27.634372 °E); site 3 is at Lake Heritage (an impoundment on the Crocodile River) (25.959696 °S, 27.855555 °E) and site 4 is in the river, below Lake Heritage (25.957086 °S, 27.858308 °E). Snails were collected from the sampling sites in summer and autumn (February–May) of 2022 and 2023. Sampling followed the procedures outlined by Outa and Avenant-Oldewage (2023). Accordingly, snails were handpicked from submerged reed stems, pebbles and boulders. Specimens were placed in 10 L plastic buckets (50 individuals per container) half-filled with water from the sampling sites. Snails were protected from direct sunlight and kept aerated by partially covering the buckets. All specimens were transferred to an onsite field laboratory, identified based on shell morphology (Craven, 1881; Walker, 1912; Connolly, 1939; Brown, 1994) and examined for digeneans within 24 h of sampling.

Screening of snails for digeneans followed the methods described by Frandsen and Christensen (1984). Digenean larvae were isolated and studied alive in temporary mounts; stained with Nile blue or unstained. Illustrations of the specimens were made with the aid of a drawing tube and digitized on Corel DRAW ® Graphics Suite X6 software (Corel Corporation, Ottawa, Canada). Ten sporocysts and 30 cercariae of each morphotype were preserved in 70% ethanol for further morphological examination. Five to 10 specimens of each morphotype (from each snail) that had been examined in unstained mounts were transferred into 96% ethanol for molecular analyses. Soft tissues of the snails (that harboured parasites) were preserved in 96% ethanol for genetic identification. For morphometric analyses, sporocysts and 20 cercariae of each morphotype were processed following the procedures of Helmer *et al*. (2023). Representative morphotypes were studied using a Zeiss Axioplan 2 epifluorescence microscope and measurements were obtained using AxioVision 4.3 imaging software (Göttingen, Germany).

Ten cercariae of each morphotype were prepared for SEM based on the methods described by Nation (1983). Representative specimens were dehydrated through 70, 80, 90, 96 and 100% ethanol. Subsequently, the specimens were transferred into a succession of 40, 70 and 100% hexamethyldisilazane (Merck, Darmstadt, Germany), for 5–7 minutes in each concentration. Specimens were mounted on adhesive conductive carbon tapes, fixed on copper stubs. Stubs with mounted specimens were placed in a Sanpla dry keeper desiccator cabinet (Kita-ku, Osaka, Japan) for 24 h. Mounted specimens were then sputter coated with gold, using an Emscope SC500 (Quorum Technologies, Newhaven, UK). Surface features of the specimens were studied using a Vega 3 LMH, Tescan (Brno, Czech Re-public) scanning electron microscope at 6 kV.

### Genetic characterization and phylogenetic analyses

For genetic analyses, specimens (preserved in 96% ethanol) were dried, and DNA was extracted using E.Z.N.A.® Tissue DNA Kit (Omega, Bio-tek, Inc, Georgia, USA), following the manufacturer's instructions. Genetic identification of the snails was based on analyses of mitochondrial CO1 gene. Partial fragments of CO1 gene were amplified using primers LCO1490 (5’-GGTCAACAAATCATAAAGATATTGG-3’) and HCO2198 (5’-TAAACTTCAGGGTGACCAAAAAATCA-3’) (Folmer *et al*., 1994), following the polymerase chain reaction (PCR) profile set by Albrecht *et al*. (2004). Digeneans were characterized using 28S, 18S and ITS rDNA. Partial sequences of 28S rDNA were amplified using primers dig12 (5′-AAGCATATCACTAAGCGG -3′) and 1500R (5′-GCTATCCTGAGGGAAACTTCG-3′) (Tkach *et al*., 2003). PCR profile (Tkach *et al*., 2003) was modified by adjusting the initial denaturation time (5 min), annealing (52°C for 1 min) and the final extension (10 min). For 18S rDNA, primers 18S-E (5’-CCGAATTCGTCGACAACCTGGTTGATCCTG CCAGT-3’) and WormB (5’-CTTGTTACGACTTTTACTTCC-3’) (Littlewood and Olson, 2001) were used, following the PCR conditions set by Králová-Hromadová *et al*. (2008). For ITS rDNA, fragments comprising of ITS1-5.8S-ITS2 were amplified using 81-f (5’-GTAACAAGGTTTCCGTAGGTGAA-3’) (Gustinelli *et al*., 2010) and ITS2.S (5’-CCTGGTTAGTTTCTTTTCCTCCGC-3’) (Cribb *et al*., 1998). PCR profile set by Gustinelli *et al*. (2010) was modified by increasing the time for initial denaturation (3 min) and annealing (45 s).

Gel electrophoresis was performed using a SmartDoc™ 2.0 ultra-violet transilluminator (Benchmark Scientific, NJ, USA). Amplification of the fragments was verified visually in 1% agarose gel loaded with SafeView™ FireRed (abm) stain. DNA sequencing was done using PCR forward and reverse primers, and the generated sequences were inspected, edited and aligned using Geneious Prime 2020.2.2, according to the procedures outlined by Kearse *et al*. (2012). Sequences were run through BLAST on GenBank, to find the nucleotide sequences with the closest similarity to the sequences generated in the present study. Sequences from the present study and those from GenBank were aligned and trimmed using MEGA7 software. Distances and differences in number of base pairs, between the aligned sequences, were compared following Tamura *et al*. (2013).

Phylogenetic analyses were performed using 28S rDNA sequences since there are more fish blood fluke representative DNA sequences, compared with 18S and ITS rDNA. Maximum likelihood (ML) and Bayesian inference (BI) methods were performed, using final alignments of 1078–1221 bp. In addition to the sequences generated from the present study, the available GenBank sequences (25) of freshwater fish blood flukes were used. Species of the family Clinostomidae: *Clinostomum complanatum* (accession no. LC483164 and MH491531) and *Euclinostomum* spp. (accession no. MW604803-06) were used as outgroup. The alignment was run through a model estimation tool on MEGA7 to select appropriate nucleotide substitution model. ML reconstruction was done in MEGA7 using general time reversal (GTR) model for nucleotide substitution, with 5 categories of discrete gamma (G) distribution. Phylogenetic BI reconstruction was done in BEAST v2.5.0 (Bouckaert *et al*., 2014) using GTR model and by applying 10 million Markov chain Monte Carlo (MCMC) analysis. In both ML and BI analyses, the estimations of nodal support values were based on 1000 bootstrap replicates.

## Results

### Prevalence and morphological descriptions

A total of 1645 specimens of *Burnupia* spp. were examined, of which 3.10% harboured furcocercariae. The cercarial characteristics: oral sucker modified into a cephalic penetration organ, apharyngeate, presence of a dorsal finfold on the body, furcae shorter than the tail stem (brevifurcate) and symmetrical, presence of dorsoventral finfolds on furcae and absence of a ventral sucker, were consistent with the members of the family Sanguinicolidae (Erickson and Wallace, 1959; Schell, 1974; Simon-Martin *et al*., 1987; Kirk and Lewis, 1993; Sendersky and Dobrovolsky, 2004; Faltýnková *et al*., 2007). Detailed morphological and molecular studies of the specimens revealed that they represented 4 species. Co-occurrence of different sanguinicolid species in individual snails was not observed. Table 1 shows the prevalence of each sanguinicolid in the hosts from the sampling sites. The digeneans have been designated Sanguinicolidae gen. sp. 1 ZA, Sanguinicolidae gen. sp. 2 ZA, Sanguinicolidae gen. sp. 3 ZA and Sanguinicolidae gen. sp. 4 ZA. Descriptions of the parthenitae and cercariae are provided below. Morphometric measurements of the cercariae for each species are presented in Table 2. All measurements are given in micrometres; presented as mean values, and the ranges are in parentheses.

**Table 1.** Prevalence (%) of sanguinicolid larvae in the examined snails from the study sites

Sampling site	Digenea	Host	Prevalence
Vaal River, below Vaal Dam (S1)	Sanguinicolidae gen. sp. 1 ZA	*Burnupia transvaalensis*	2.54
	Sanguinicolidae gen. sp. 2 ZA	*B*. *transvaalensis*	1.19
Vaal Rive, below Vaal Barrage (S2)	Sanguinicolidae gen. sp. 1 ZA	*B*. *transvaalensis*	1.52
		*Burnupia mooiensis*	1.01
	Sanguinicolidae gen. sp. 2 ZA	*B*. *transvaalensis*	0.76
		*B*. *mooiensis*	1.01
Lake Heritage (S3)	Sanguinicolidae gen. sp. 3 ZA	*Burnupia trapezoidea*	7.81
	Sanguinicolidae gen. sp. 4 ZA	*B*. *trapezoidea*	0.78
Crocodile River, below L. Heritage (S4)	Sanguinicolidae gen. sp. 3 ZA	*B*. *trapezoidea*	2.02

*B*. *transvaalensis*: S1 (*n* = 590), S2 (*n* = 132); *B*. *mooiensis*: S2 (*n* = 398); *B*. *trapezoidea*: S3 (*n* = 128), S4 (*n* = 397).

**Table 2.** Cercarial measurements (in *μ*m) of the current species (in bold) and the previously described species of the family Sanguinicolidae

Species	Body length	Body width	Cephalic organ length	Cephalic organ width	Tail stem length	Tail stem width	Furcal length	Furcal base width
**Sanguinicolidae gen. sp. 1 ZA**	142 (135-147)	44 (41–51)	24 (23–25)	18 (16–20)	250 (238–270)	29 (25–34)	94 (89–98)	12 (10–13)
**Sanguinicolidae gen. sp. 2 ZA**	160 (144–181)	43 (41–48)	25 (22–29)	20 (18–23)	251 (241–261)	31 (27–36)	82 (75–87)	12 (10–14)
**Sanguinicolidae gen. sp. 3 ZA**	132 (122–147)	35 (33–40)	25 (23–26)	18 (16–20)	225 (212–234)	30 (29–33)	73 (70–76)	13 (12–15)
**Sanguinicolidae gen. sp. 4 ZA**	141 (123–169)	35 (31–43)	23 (20–26)	17 (16–18)	227 (222–232)	30 (28–30)	67 (58–75)	10 (9.4–11)
^a^*Cercaria sewelli*	150	48			180		70	
^b^*Cercaria britspennata*	67–131	20–28	13–20	12–15	87–180		33–50	
^b^*Cercaria capensis*	110–130	35–46	20–30	16–22	185–256		50–86	
^c^*Sanguinicola cristafer*	202–234	52–60	31–34	27–31	320–347	35–48	118–139	
^c^*S. lophophora*	115–135	28–34	28–31	17–21	245–275	19–26	93–109	
^d^*S. idahoensis*	107–124	36–42			271–327	28–30	64–78	
^e^*S. lungesnsis*	150				260		90	
^f^*S. rutili*	114	34	19		228	20	79	
^g^*S. inermis*	96 (83–108)	29 (25–33)	16 (13–20)		199 (190–208)	18 (17–20)	76 (66–93)	
^h^*S. armatus*	90–100	27 (20–30)	15 (10–20)		280 (260–290)	22 (20–25)	60–70	
^i^*Sanguinicola* sp.	133 (125–145)	41 (38–48)	28 (25–30)	25 (23–28)	245 (215–263)	29 (28–38)	72 (63–93)	15 (10–18)

^a^Faust (1926); ^b^Porter (1938); ^c^Erickson and Wallace (1959); ^d^Schell (1974); ^e^Tang and Ling (1975); ^f^Simon-Martin *et al*. (1987); ^g^Kirk and Lewis (1993); ^h^Sendersky and Dobrovolsky (2004); ^i^Faltýnková *et al*. (2007).

#### Sanguinicolidae gen. sp. 1 ZA

Localities: Vaal River, below Vaal Dam and below Vaal Barrage (Orange River system) South Africa.

Host: *Burnupia transvaalensis* (Craven, 1881) and *B. mooiensis* (Walker, 1912) (Gastropoda: Burnupiidae).

Genetic material: Accession numbers of the representative rDNA sequences; 28S (OR892787, OR892788 and OR892791), 18S (OR892796 and OR892797) and ITS (OR892801-OR892804).

**Figure 2.**
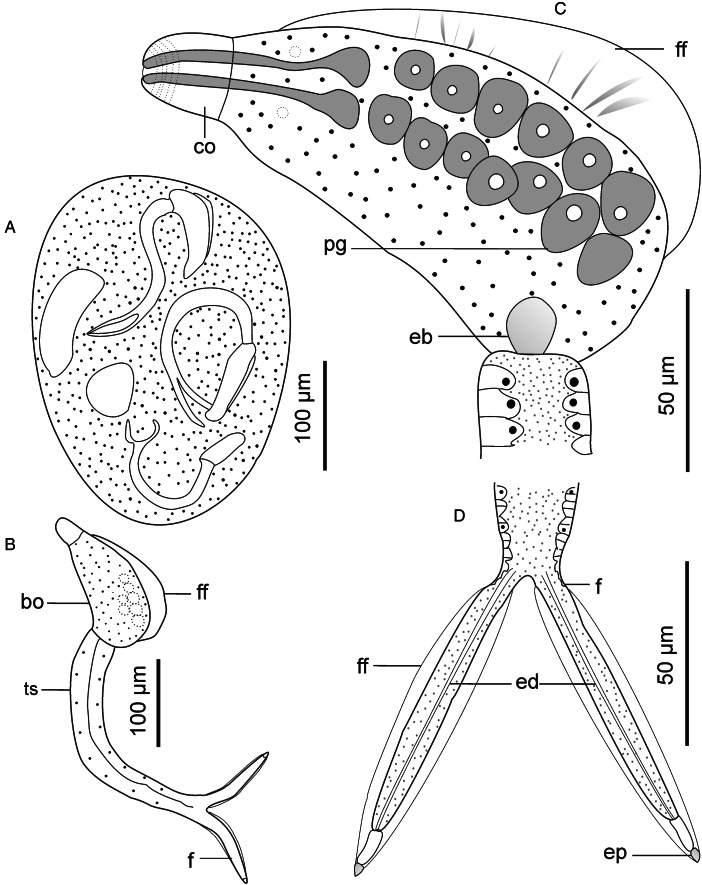
Schematic drawings of Sanguinicolidae gen. sp. 1 ZA. A, sporocyst; B, whole cercaria; C, cercarial body and D, furcae. Abbreviations: bo, body; co, cephalic organ; eb, excretory bladder; ed, excretory duct; ep, excretory pore; f, furca; ff, finfold; pg, penetration gland and ts, tail stem.

**Figure 3.**
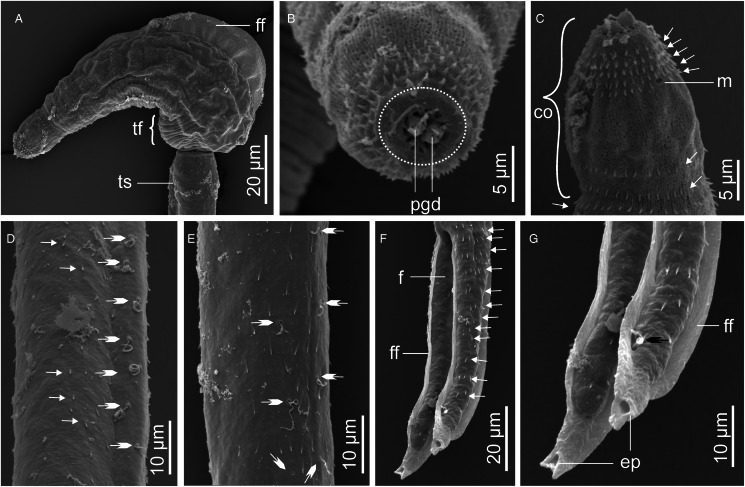
Scanning electron micrographs of Sanguinicolidae gen. sp. 1 ZA cercaria. A, cercarial body; B, enface view of anterior end showing circlet of papillae (inside the broken line circle); C, ventral view of anterior end; D, latero-dorsal view of mid region of tail stem; E, subventral view of the mid region of tail stem; F, lateral view of furcae and G, posterior end of furcae. Triangle arrow heads show rows of spines and winged arrows show papillae with sensilla. Abbreviations: co, cephalic organ; ep, excretory pore; f, furca; ff, finfold; m, mouth; pgd, tip of penetration gland ducts; tf, transverse folds and ts, tail stem.

Descriptions: Sporocysts found embedded in digestive gland. Sporocysts ovoid, 275 (251–301) × 194 (178–214), contain 3–4 developed cercariae (Fig. 2A). Cercarial body curved ventrad, bearing dorsal finfold on convex side (Fig. 2C and 3A). Finfold pleated and aspinous, visible in live stained and unstained specimens, indistinct in some fixed specimens. Finfold originates in second quarter of body, extends over 68 (60–74)% of dorsal body surface; height, 28 (25–31)% of body width. Anterior part of body modified into ovate cephalic penetration organ, bearing protrusible tip. Transverse constriction of tegument separates cephalic organ from main body. Numerous cystogenous glands obscure internal body structures. Mouth opening discernible using SEM, subapical, located on ventral side of cephalic organ (Fig. 3C). Digestive tube indistinct; pharynx and ventral sucker absent. A pair of round structures resembling non-pigmented eyespots present posterior to apical organ on either side of body in some specimens. Six to 8 (usually 7) pairs of penetration glands visible in stained and unstained specimens (Fig. 2C). Ducts from penetration glands extend laterally to cephalic organ apex. Excretory bladder present at posterior end of body. Excretory ducts undiscernible on tail stem, visible in each furca, open at furcal tip (Fig. 2D and 3G). Body tegument characterized by prominent dorsal and lateral longitudinal ridges. Ventral transverse folds present near posterior end of body (Fig. 3A). Tegument spinous; spines arise from furrows between longitudinal ridges. Spines pointed posteriorly, arranged in 32–35 concentric rows from anterior end to about 70% of body length. Cephalic organ bears 7 circlets of spines: 5 on anterior part, 2 on posterior part and mid region devoid of spines (Fig. 3C). Anterior circlets of cephalic organ closely spaced, bear robust, sharp spines; posterior circlets widely spaced (3–4 times wider than anterior circlets), bear slender spines. Tail stem straight, 1.8 (1.7–1.9) times longer than body. Dorsal longitudinal furrow extends posteriorly from tail base to about 70% of stem length. Tail stem bears posteriorly pointed slender spines and sensilla that arise from dorso-ventrally located papillae (Fig. 3D and E). Furcae, 2.6 (2.4–3.0) times shorter than tail stem; bear dorso-ventral finfolds. Finfolds indistinct in some fixed specimens when observed with light microscope. Each furca has 12–13 transverse rows of spines on lateral sides, from base to about 80% of furcal length (Fig. 3F). Furcal tip tapered, 18 (16–20) long, with 2 transverse constrictions (Fig. 2D); latero-dorsal sensillum present near tip (Fig. 3G).

#### Remarks

The cercaria differs from the other cercarial types from the current study (see Sanguinicolidae gen. sp. 2 ZA, Sanguinicolidae gen. sp. 3 ZA and Sanguinicolidae gen. sp. 4 ZA, described below) based on longer body finfold, relative to the body; longer furcae; more penetration glands; fewer spine-bearing circlets on the cephalic penetration organ and absence of spines in middle part of the cephalic organ. Compared with previous studies from Africa, the present cercaria resembles *Cercaria capensis* which was described by Porter (1938) from *Bur*. *capensis* in Oudtshoorn, South Arica. However, *C. capensis* is produced in oblong and large sporocysts (350–500 long, 50–90 wide); different to the present species whose sporocysts are ovoid and smaller (251–301 long, 178–214 wide). What is more, the finfold of *Ce. capensis* extends from the cephalic organ to near the posterior end of the body (Porter, 1938, Plate LXXVII, Fig. 3). In the present cercaria, the finfold originates posterior to the cephalic penetration organ and covers approximately three-quarters of the body length. Based on the features of the dorsal finfold and the numbers of large gland cells in the body, the present cercaria resembles 4 other digeneans from North America and Europe. These are: *Sanguinicola idahoensis* Schell, 1974 from *Fluminicola virens* (Lea, 1838) (Lithoglyphidae), *Sanguinicola lophophora* Erickson and Wallace, 1959 and *Sanguinicola cristafer* Erickson and Wallace, 1959 from *Valvata* spp. (Valvatidae) in the USA; *Sanguinicola armatus* Plehn, 1905 from *Lymnaea stagnalis* (Linnaeus, 1758) and *Sanguinicola* sp. from *Valvata macrostoma* Mörch, 1864 in Finland (Faltýnková *et al*., 2007). Indeed, the numbers of large gland cells in the body are nearly identical between the species: 6–9 pairs in *S*. *lophophora* and *S*. *cristafer* (Erickson and Wallace, 1959), at least 6 pairs in *S*. *idahoensis* (Schell, 1974), 8 pairs in *S*. *armatus* (Sendersky and Dobrovolsky, 2004), 8–11 pairs in *Sanguinicola* sp. (Faltýnková *et al*., 2007) and 6–8 pairs in the current specimens. However, unlike in the current specimens where the excretory bladder is nearly round, the excretory bladder is bilobed in *S*. *idahoensis* (Schell, 1974), V-shaped in *S. armatus* (Sendersky and Dobrovolsky, 2004) and Y-shaped in *Sanguinicola* sp. (Faltýnková *et al*., 2007). *Sanguinicola lophophora* and *S*. *cristafer* are distinguished by the presence of long dorso-ventral caudal hairs (observed in living cercariae); a feature that was not observed on the cercaria from the present study. Finally, the present cercaria is distinguished from the other digeneans based on body length, relative to the dimensions of the tail stem and furcae (Table 2).

#### Sanguinicolidae gen. sp. 2 ZA

Localities: Vaal River, below Vaal Dam and below Vaal Barrage (Orange River system) South Africa.

Host: *Burnupia transvaalensis* (Craven, 1881) and *B. mooiensis* (Walker, 1912) (Gastropoda: Burnupiidae).

Genetic material: Accession numbers of the representative rDNA sequences; 28S (OR892789 and OR892792), 18S (OR892800 and OR892805) and ITS (OR896076).

**Figure 4.**
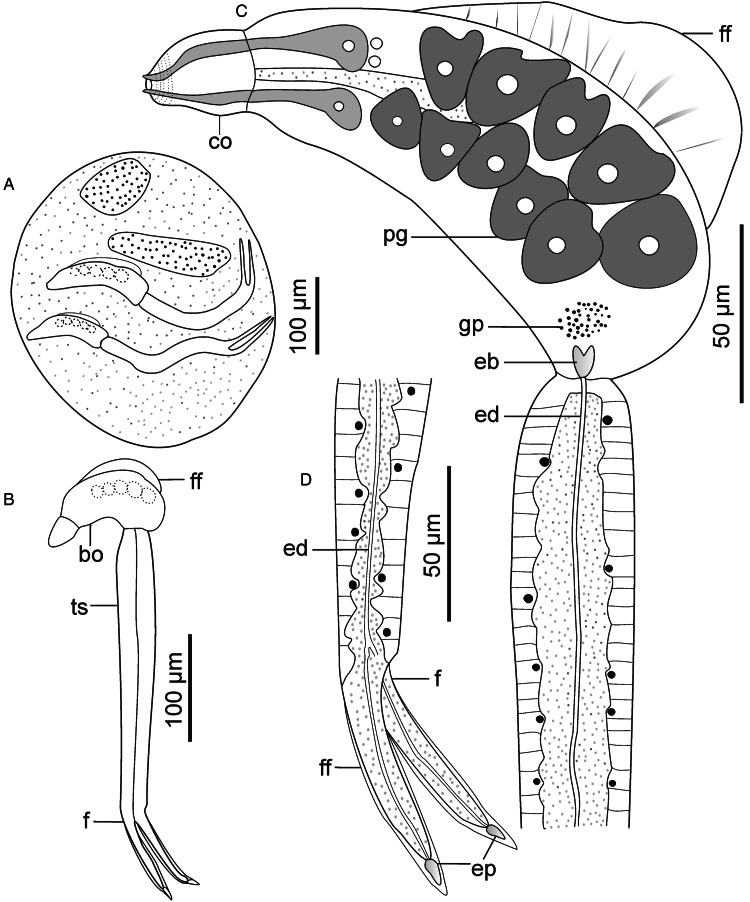
Schematic drawings of Sanguinicolidae gen. sp. 2 ZA. A, sporocyst; B, whole cercaria; C, cercarial body and D, posterior end of tail. Abbreviations: bo, body; co, cephalic organ; eb, excretory bladder; ed, excretory duct; ep, excretory pore; f, furca; ff, finfold; gp, genital primordium; pg, penetration gland and ts, tail stem.

**Figure 5.**
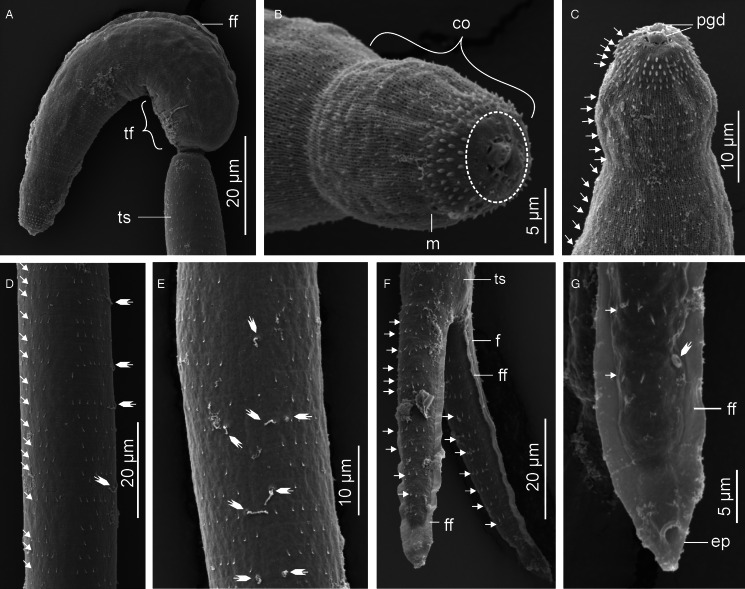
Scanning electron micrographs of Sanguinicolidae gen. sp. 2 ZA cercaria. A, cercarial body; B, lateral view of anterior end showing circlet of papillae (inside the broken line circle); C, dorsal view of anterior end; D, latero view of mid region of tail stem; E, up-close view of the ventral side (mid region) of tail stem; F, lateral view of furcae and G, furcal posterior end. Triangle arrow heads show rows of spines and winged arrows show papillae with sensilla. Abbreviations: co, cephalic organ; ep, excretory pore; f, furca; ff, finfold; m, mouth; pgd, tip of penetration gland ducts; tf, transverse folds and ts, tail stem.

Descriptions: Sporocysts found buried in digestive gland; difficult to separate from host's tissues. Sporocysts ovoid, 418 (394–438) × 315 (302–334); bearing 2–3 germ balls and 2 developed cercariae (Fig. 4A). Cercarial body curved ventrally, bearing pleated, aspinous dorsal finfold (Fig. 4C). Finfold originates in second third of body, extends over 55 (50–61)% of dorsal body length; height, 47 (41–53)% of body width. Finfold observable in live specimens, nearly invisible in some preserved specimens when examined optically. Anterior part of body modified into pear-shaped cephalic penetration organ bearing protrusible tip. Cephalic organ differentiated from main body by transverse constriction of tegument. Mouth opening, visible with SEM, subapical, located on ventral side of cephalic organ (Fig. 5B). Intestine-like tube extends from posterior part of cephalic organ to about half of body length. Pharynx and ventral sucker absent. Body bears a pair of poorly visible glands posterior to cephalic organ and 5 prominent pairs of penetration glands, arranged along body length, covering nearly same distance as body finfold (Fig. 4C). Genital primordium comprises of discoid mass of cells between posterior pair of penetration glands and excretory bladder (Fig. 4C). Excretory duct extends from excretory bladder, bifurcates at posterior end of tail stem, into 2 canals that terminate at lateral pores on furcal tips (Fig. 4C, D and 5G). Tegument bears well-defined longitudinal ridges, papillae and spines. Apex of cephalic organ lacks spines: bears circlets of ciliated and sheathed unciliated papillae (Fig. 5B). Tegumental spines pointed posteriorly, arranged in 34–35 concentric rows from anterior to about two-thirds of body. Cephalic organ bears 11 circlets of spines: anterior 5 circlets densely spaced, consist of robust spines; posterior 6 circlets widely spaced, about 2 times wider apart than anterior circlets, bear slender spines, like spines on main body (Fig. 5B and C). Tail stem straight, 1.6 (1.3–1.7) times longer than body; bears posteriorly pointed slender spines and sensilla that arise from dorso-ventral papillae (Fig. 5D and E). Furcae 3.1 (2.9–3.4) times shorter than tail stem, surrounded by dorso-ventral finfolds. Furcal finfolds visible in live specimens, indistinct in some fixed specimens when observed using light microscope. Lateral sides of furcae bear slender, sharp spines, densely arranged in 11–12 transverse rows, from base to about three-quarters of furcal length (Fig. 5F). Single latero-dorsal sensillum present near tip of each furca (Fig. 5G); furcal tip attenuated, 12 (10–14) long.

#### Remarks

Cercaria of Sanguinicolidae gen. sp. 2 ZA differs from the other species in the present study based on its longer body (Table 2). Also, it bears 5 pairs of penetration glands, which is fewer than in the above species and more in number than in the 2 species described below. Finally, it bears 11 rows of spines on the cephalic organ, which is higher in number than in Sanguinicolidae gen. sp. 1 and Sanguinicolidae gen. sp. 4, but less than in Sanguinicolidae gen. sp. 3. Based on the presence of 5 prominent pairs of penetration glands, the present specimens resemble *Cercaria indicanonoides* reported by Porter (1938), from the bulinid, *Bulinus africanus* (Krauss, 1848) in Sydenham, near Durban, South Africa and *Sanguinicola rutili* from a planorbid, *Ancylus fluviatilis* Müller, 1774 in Spain (Simon-Martin *et al*., 1987). The present species is characterized by large sporocysts (394–438 long), which are about double the size of mature sporocysts of *C*. *indicanonoides* (102–220 long) (Porter, 1938) and *S*. *rutili* (190–320 long) (Simon-Martin *et al*., 1987). Secondly, *C. indicanonoides* is ocellate, a feature that distinguishes it from the present cercaria. Also, Sanguinicolidae gen. sp. 2 SA has a shorter finfold that originates in the second third of the body and terminates before the posterior end. In *C*. *indicanonoides*, the finfold originates at the constriction that demarcates the cephalic organ and extends to the posterior end of the body (Porter, 1938, Plate LXXX, Fig. 3). *Sanguinicola rutili* is distinguished by numerous long setae on the caudal stem, which are absent in the present specimens. Finally, *S*. *rutili* has a smaller body and shorter tail stem compared with the present cercaria (Table 2).

#### Sanguinicolidae gen. sp. 3 ZA

Localities: Lake Heritage and below Lake Heritage, Crocodile River (Limpopo River system), South Africa.

Host: *Burnupia trapezoidea* (Boettger, 1910) (Gastropoda: Burnupiidae).

Genetic material: Accession numbers of the representative rDNA sequences; 28S (OR892790, OR892793 and OR892794), 18S (OR900600) and ITS (OR896075, OR896077 and OR896078).

**Figure 6.**
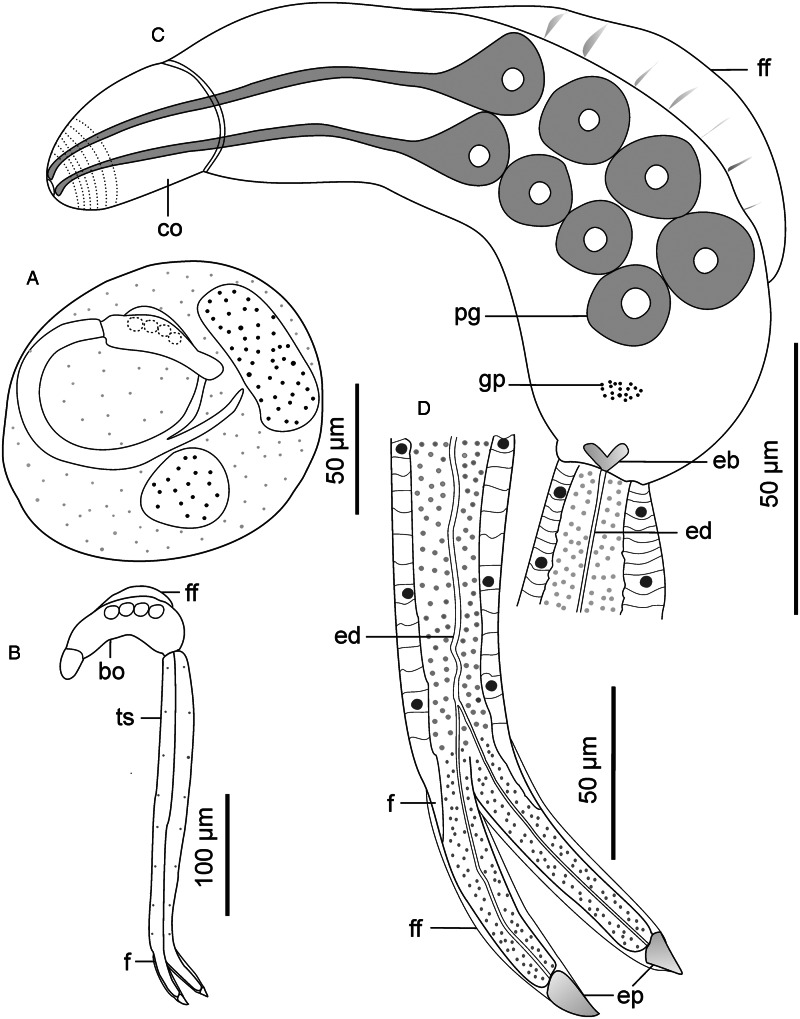
Schematic drawings of Sanguinicolidae gen. sp. 3 ZA. A, sporocyst; B, whole cercaria; C, cercarial body and D, posterior end of tail. Abbreviations: bo, body; co, cephalic organ; eb, excretory bladder; ed, excretory duct; ep, excretory pore; f, furca; ff, finfold; gp, genital primordium; pg, penetration gland and ts, tail stem.

**Figure 7.**
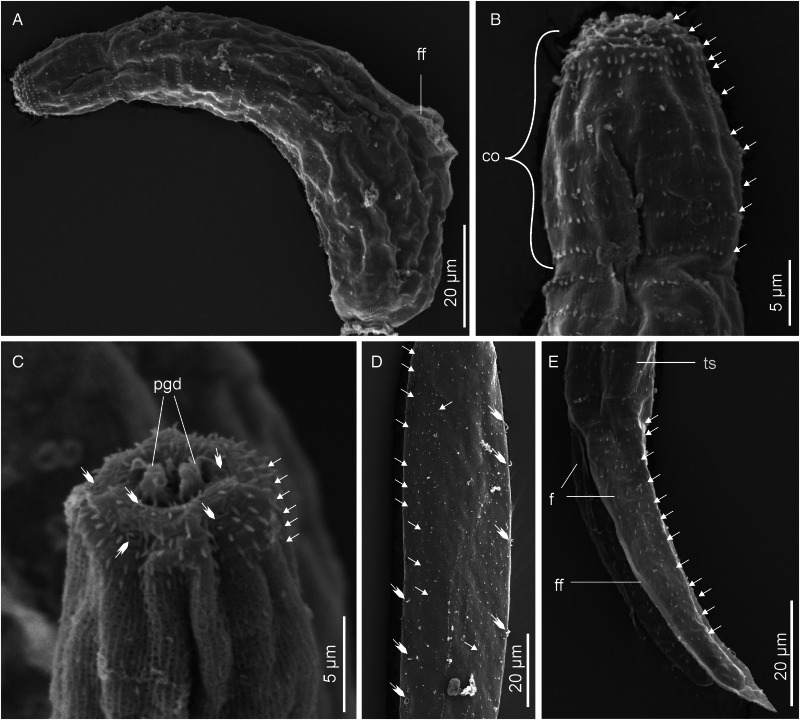
Scanning electron micrographs of Sanguinicolidae gen. sp. 3 ZA cercaria. A, cercarial body; B, lateral view of the cephalic penetration organ; C, apical view of anterior end; D, latero view of the anterior half of tail stem; E, lateral view of furcae. Triangle arrow heads show rows of spines and winged arrows show papillae with sensilla. Abbreviations: co, cephalic organ; f, furca; ff, finfold; tip of penetration gland ducts and ts, tail stem.

Descriptions: Sporocysts nearly spherical, 118 (109–132) × 110 (98–121), contain 1 developed cercaria and 2 germ balls (Fig. 6A). Cercarial body curved ventrad; convex side bears pleated, aspinous finfold (Figs 6C and 7A). Finfold originates in second third of body, extends over 48 (41–52)% of dorsal body length; height, 47 (41–53)% of body breadth. Body finfold undiscernible in some preserved specimens when viewed using light microscope. Anterior part of body modified into ovoid cephalic organ bearing protrusible tip. Cephalic organ demarcated from main body by transverse constriction of tegument. Pharynx and ventral sucker absent; digestive tube not observed. Body bears 4 pairs of penetration glands with ducts that open at anterior end of cephalic organ (Fig. 6C). Genital primordium consists of aggregation of cells located between last pair of penetration glands and excretory bladder. Excretory bladder opens into posterior duct that extends along tail stem, bifurcates into canals that terminate at furcal tips (Fig. 6C and D). Body surface characterized by network of fine transverse and prominent longitudinal ridges. Tegumental spines pointed posteriorly, arranged in 29–31 concentric rows, from anterior to approximately two-thirds of body length. Cephalic organ bears 12 transverse rows of spines: first 3 circlets have papillae interspersed between spines (Fig. 7C). Spines on anterior 6 rows of cephalic organ, robust, blunt and densely arranged; posterior rows widely spaced (2–3 times wider than on anterior part), bear slender spines (Figs 7B and C). Tail stem straight, 1.7 (1.6–1.9) times longer than body. Tail stem spinous, with papillae bearing sensilla, arranged in dorsoventral longitudinal rows (Fig. 7D). Furcae laterally flattened, 3.1 (2.8–3.1) times shorter than tail stem; surrounded by narrow dorso-ventral finfolds (Fig. 6D and 7E). Lateral surfaces of the furcae bear 12–13 transverse rows of spines, covering approximately three-quarters of furcal length (Fig. 7E). The furcal tip tapered, 9.6 (8.6–11) long.

#### Remarks

Sanguinicolidae gen. sp. 3 ZA is distinguished from the other species from the current study by small-sized sporocysts that are nearly spherical. Its cercaria has 4 pairs of penetration glands, which is fewer than in the 2 species described above, and more than in Sanguinicolidae gen. sp. 4 ZA. Finally, it is characterized by an ovoid cephalic organ that bears 12 circlets of spines, which is more than in the other 3 species. Based on the number of penetration glands, the present cercaria resembles *Cercaria britspennata* Porter, 1938 that was found in thiarid *Melanoides tuberculata* (Müller, 1774) from the Crocodile River, South Africa (Porter, 1938), and *S*. *inermis* reported by Kirk and Lewis (1993) from lymnaeid *Peregriana peregra* (Müller, 1774) in England. Sanguinicolidae gen. sp. 3 ZA has a much shorter dorsal finfold that covers 0.41–0.52 of the body length, compared with *C*. *britspennata* whose finfold is about 0.80 of the body length (Porter, 1938, Plate LXXVIII, Fig. 3). According to Kirk and Lewis (1993), SEM images of *S*. *inermis* revealed 5 rows of thick spines on the middle region of the cephalic organ. In contrast, the cephalic organ of the current cercaria bears 12 circlets of spines; the anterior 6 rows have thick spines, followed by 7 posterior rows of thin spines. Finally, both *C*. *britspennata* and *S*. *inermis* are smaller in size, compared to the present cercaria (Table 2).

#### Sanguinicolidae gen. sp. 4 ZA

Localities: Lake Heritage, Crocodile River (Limpopo River system), South Africa.

Host: *Burnupia trapezoidea* (Boettger, 1910) (Gastropoda: Burnupiidae).

Genetic material: Accession numbers of the representative rDNA sequences; 28S (OR892795) and 18S (OR892798 and OR892799).

**Figure 8.**
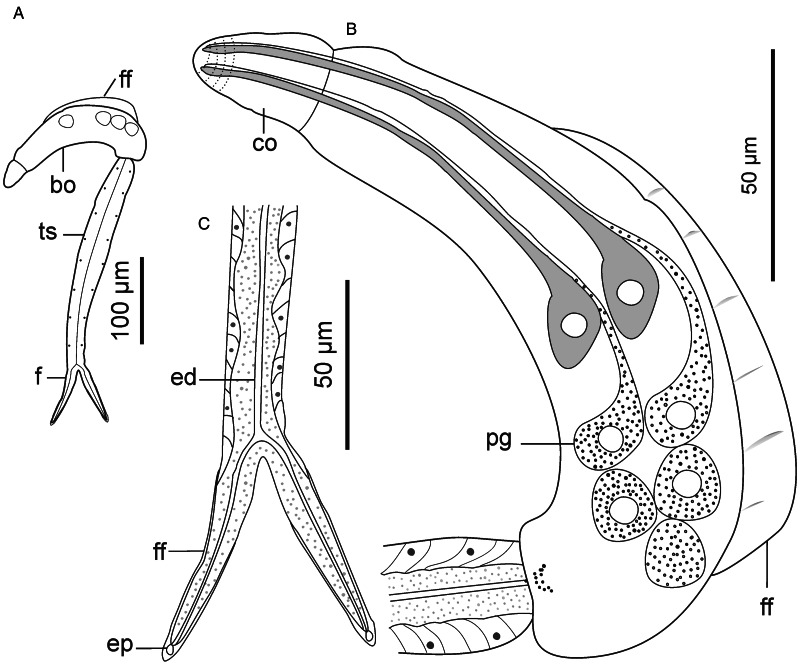
Schematic drawings of Sanguinicolidae gen. sp. 4 ZA. A, whole cercaria; B, cercarial body and C, posterior end of tail. Abbreviations: bo, body; co, cephalic organ; ed, excretory duct; ep, excretory pore; f, furca; ff, finfold; pg, penetration gland and ts, tail stem.

**Figure 9.**
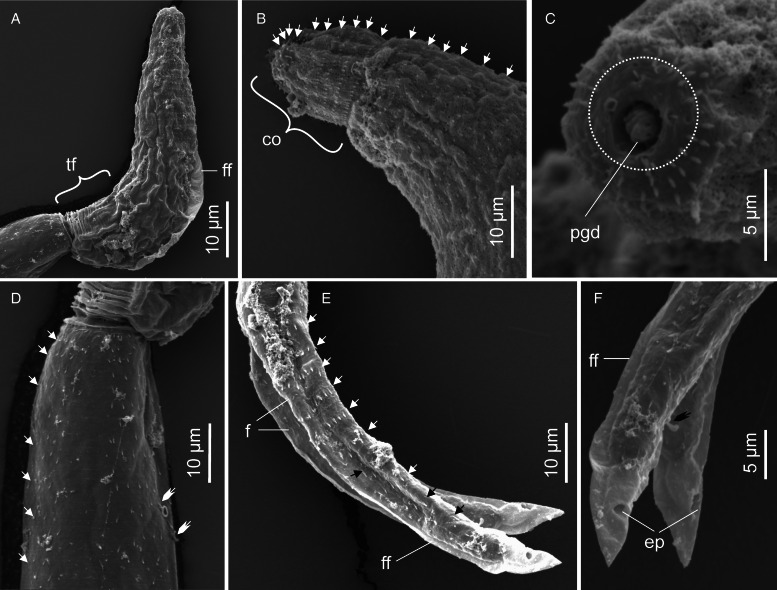
Scanning electron micrographs of Sanguinicolidae gen. sp. 4 ZA cercaria. A, cercarial body; B, lateral view of anterior end; C, enface view of anterior end showing circlet of papillae (inside the broken line circle); D, lateral view of anterior region of tail stem; E, lateral view of furcae and F, furcal posterior end. Triangle arrow heads show rows of spines and winged arrows show papillae with sensilla. Abbreviations: co, cephalic organ; ep, excretory pore; f, furca; ff, finfold; pgd, tip of penetration gland ducts; tf, transverse folds and ts, tail stem.

Descriptions: Cercariae found from 1 snail specimen; sporocysts not observed. Cercarial body curved ventrally, dorsal side bears pleated, aspinous finfold (Fig. 8B and 9A). Finfold originates from second third of body, extends over 51 (48–53)% of dorsal body surface; height, 30 (27–32)% of body width. Finfold indistinct in most preserved specimens when examined optically. Anterior end bears ovate cephalic organ, demarcated from main body by transverse constriction of tegument. Pharynx, digestive tube and ventral sucker not observed. Three pairs of prominent gland cells: an equatorial pair and 2 pairs located in posterior half of body (Fig. 8B). Excretory canal runs through tail stem and furcae, open externally through latero-dorsal pores near furcal tips (Fig. 8C and 9F). Tegumental spines pointed posteriorly, occur in 25 concentric rows around body, covering about 40% of body length from anterior end. Rim of the cephalic organ bears sheathed papillae that have sensilla (Fig. 9C). Cephalic organ has 10 circlets of spines (Fig. 9B). First 4 circlets on cephalic organ are closely arranged and bear robust spines; posterior 6 circlets widely spaced (2.5–3.0 times further apart than anterior rows) and consist of slender spines that resemble those on the main body. Tail stem 1.6 (1.4–1.7) times longer than body; spinous and bears papillae with sensilla, arranged in longitudinal dorsoventral rows (Fig. 9D). Furcae 3.6 (3.1–3.9) times shorter than tail stem. Each furca bears narrow finfold that extends dorsally along furcal length, over the tip and cover only distal half on ventral side (Fig. 8C and 9E); finfold poorly visible in preserved specimens when examined using light microscope. Furcal spines arranged in 10–12 transverse rows on lateral sides of each furca, from base to about three-quarters of furcal length. Furcal tip tapered, 10.1 (8.94–11.6) long.

#### Remarks

This species is distinguished from the other species in the current study by fewer rows of spines (25) that cover less than half of the body length. In contrast, spines cover at least two-thirds of the body length in the other 3 species. Secondly, Sanguinicolidae gen. sp. 4 ZA is characterized by shorter furcal finfolds than in the other species. What is more, it has a unique arrangement of penetration glands, consisting of an equatorial pair and the rest occurring towards the posterior end of the body. The gross morphology of the current cercaria closely resembles *Cercaria sewelli* Faust, 1926. *Cercaria sewelli* was reported in *Bur*. *capensis* from Kwazulu Natal, *Bur. trapezoidea* from Free State and *Bulinus tropicus* (Krauss, 1848) from Gauteng, South Africa (Faust, 1926; Porter, 1938). Like *C*. *sewelli*, the present cercaria has a tail stem that is only slightly longer than the body and possesses short furcae that are covered with very thin finfolds (Faust, 1926; Porter, 1938). In addition, the body bears 3 pairs of prominent gland cells: an equatorial pair and 2 pairs located in the distal half (Faust, 1926, Plate VI, Fig. 1). However, Faust (1926) considered the anterior pair of glands to be large eyespots. What is more, while the furcae of the present cercaria are nearly similar in length to *C*. *sewelli*, the body and tail stem of the latter are shorter (Table 2). Since SEM images and genetic characteristics of *C*. *sewelli* are unavailable, we cannot confirm if it is identical to Sanguinicolidae gen. sp. 4 ZA.

### Genetic data and phylogenetic results

New partial sequences for 28S and 18S rDNA genes were successfully generated from at least 5 representative samples for the 4 sanguinicolids described above. For ITS rDNA, usable sequences were obtained for all the morphotypes, except for Sanguinicolidae gen. sp. 4 SA. The newly generated 28S rDNA (1117–1161 bp) showed intraspecific variation of between 2 and 3 base pair differences. Blast results from GenBank showed that the present specimens had the highest genetic similarity with an isolate presumed to be *Sanguinicola* cf. *inermis* (accession no. AY222180.1; AY222098.1). Alignment of the 28S rDNA sequences obtained in the current study with ‘*Sanguinicola* cf. *inermis*’ showed that p-distances varied between 2.87 and 7.24%, corresponding to 32–92 nucleotide substitutions (Supplementary file 2). ‘Sanguinicolid’ spp. from East Africa (Brant *et al*., 2006) differed from the present specimens by a p-distance range of 21.5–25.0%. In general, the present specimens and ‘*S*. cf. *inermis*’ showed a high genetic divergence (11.9–26.9%) from other freshwater fish blood flukes for which 28S rDNA sequences are available (Supplementary file 2). Analyses of the 18S rDNA sequences from the current study showed that intraspecific variation did not exceed 1 nucleotide substitution. For 18S rDNA, alignment of the present sequences (1745–1766 bp) with ‘*S.* cf. *inermis*’ revealed that genetic divergence between the species ranged from 2.2 to 4.0%, corresponding to 21–67 base pair differences (Table 3). ‘Sanguinicolid’ spp. from East Africa (Brant *et al*., 2006) differed from the present specimens by 11.8–14.1% (Table 3). Based on ITS rDNA (1045–1245 bp) analyses, p-distances ranged from 5.60 to 10.4%, between the present species (Table 4). There are no ITS rDNA sequences for sanguinicolids, hence, the sequences generated from the current study provide data for future genetic comparisons.

**Table 3.** The number of base pair differences (below the diagonal) and sequence divergence (%) (above the diagonal), of the present specimens (in bold) and other freshwater blood flukes, based on 18S rDNA analyses

S/N	Accession no.	Species	1	2	3	4	5	6	7	8	9	10	11	12	13
1	OR892796	**Sanguinicolidae gen. sp. 1 ZA**	**–**	**0.0**	**3.2**	**3.3**	**3.6**	**3.6**	**3.9**	**3.8**	2.6	11.8	12.0	12.7	13.9
2	OR892797	**Sanguinicolidae gen. sp. 1 ZA**	**0.0**	**–**	**3.2**	**3.3**	**3.6**	**3.6**	**3.9**	**3.9**	2.6	11.8	11.9	12.6	13.7
3	OR892800	**Sanguinicolidae gen. sp. 2 ZA**	**54**	**54**	**–**	**0.1**	**2.5**	**2.5**	**2.2**	**2.1**	3.3	12.6	12.3	12.8	14.1
4	OR892805	**Sanguinicolidae gen. sp. 2 ZA**	**55**	**54**	**1.0**	**–**	**2.6**	**2.6**	**2.2**	**2.2**	3.4	12.7	12.4	12.9	14.1
5	OR900600	**Sanguinicolidae gen. sp. 3 ZA**	**61**	**60**	**42**	**43**	**–**	**0.0**	**2.9**	**2.9**	3.8	12.5	12.2	12.8	13.7
6	OR900600	**Sanguinicolidae gen. sp. 3 ZA**	**61**	**60**	**42**	**43**	**0.0**	**–**	**2.9**	**2.9**	3.8	12.5	12.2	12.8	13.7
7	OR892798	**Sanguinicolidae gen. sp. 4 ZA**	**65**	**65**	**36**	**37**	**49**	**49**	**–**	**0.1**	3.3	12.6	13.0	13.4	13.9
8	OR892799	**Sanguinicolidae gen. sp. 4 ZA**	**65**	**65**	**35**	**36**	**48**	**48**	**1.0**	**–**	3.3	12.6	12.9	13.4	13.9
9	AY222098	‘*Sanguinicola* cf. *Inermis*’	44	44	56	57	63	63	56	56	–	12.2	12.7	12.7	13.8
10	AY829250	‘Sanguinicolid’ sp. W5003	199	198	210	211	210	210	212	213	206	–	11.8	12.3	12.5
11	AY829253	‘Sanguinicolid’ sp. W1134	202	199	205	206	204	204	217	217	214	199	–	4.6	10.7
12	AY829254	‘Sanguinicolid’ sp. W1284	214	211	213	214	214	214	225	225	213	208	78	–	10.3
13	AY829251	‘Sanguinicolid’ sp. W5004	233	229	234	234	228	228	233	233	232	210	181	174	–

**Table 4.** The number of base pair differences (below the diagonal) and sequence divergence (%) (above the diagonal), of the present sanguinicolids, based on ITS rDNA analyses

S/N	Accession no.	Species	1	2	3	4	5	6	7	8
1	OR892801	Sanguinicolidae gen. sp. 1 ZA	–	0.0	0.0	0.1	6.8	8.7	8.7	8.7
2	OR892802	Sanguinicolidae gen. sp. 1 ZA	0.0	–	0.0	0.3	7.8	10.3	8.7	10.2
3	OR892803	Sanguinicolidae gen. sp. 1 ZA	0.0	0.0	–	0.0	7.4	9.8	8.6	9.8
4	OR892804	Sanguinicolidae gen. sp. 1 ZA	1.0	4.0	0.0	–	7.7	10.4	9.0	10.2
5	OR896076	Sanguinicolidae gen. sp. 2 ZA	65	78	71	77	–	6.6	5.6	6.4
6	OR896075	Sanguinicolidae gen. sp. 3 ZA	88	111	101	111	67	–	0.1	0.0
7	OR896077	Sanguinicolidae gen. sp. 3 ZA	88	88	84	91	55	1.0	–	0.1
8	OR896078	Sanguinicolidae gen. sp. 3 ZA	88	108	101	108	64	0.0	1.0	–

**Figure 10.**
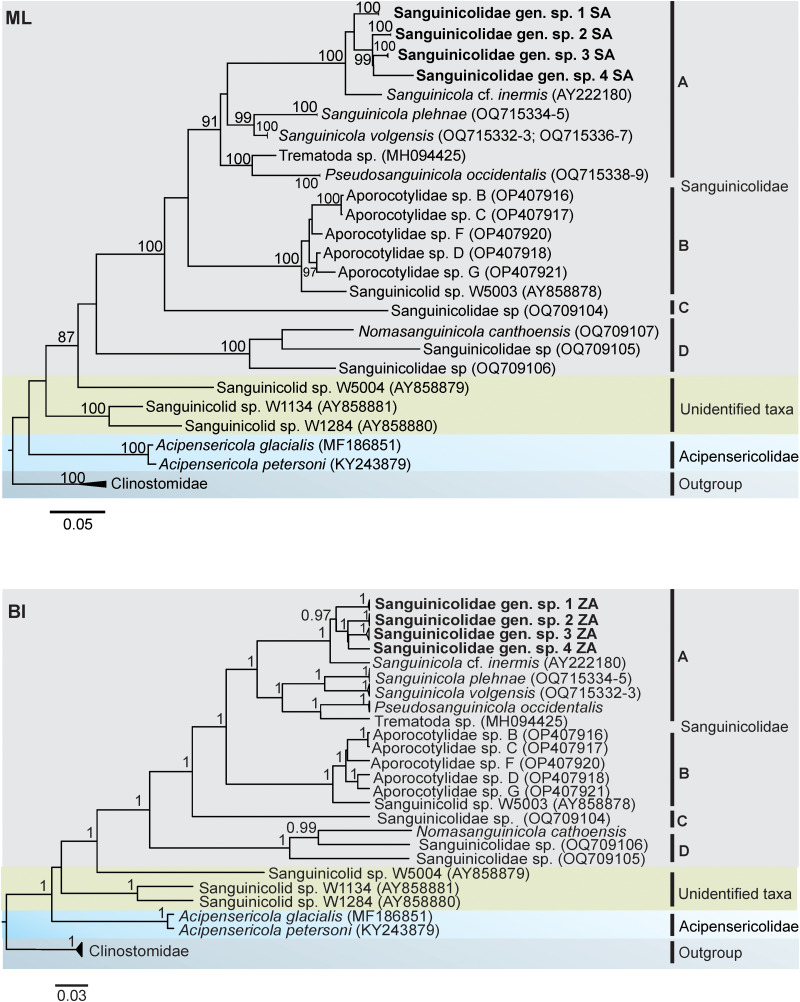
Maximum likelihood (ML) and Bayesian inference (BI) reconstructions of the phylogenetic relationships between the present species (in bold) and other blood flukes of freshwater fish, based on 28S rDNA data. The branch length scale indicates the number of substitutions per site. Nodal support values that are below 80/0.8 have been excluded.

The general topology of the phylogenetic trees was similar with that of fish blood flukes from previous studies (Cutmore *et al*., 2023; Warren and Bullard, 2023). In both the BI and ML phylograms, sanguinicolids were monophyletic and formed 4 distinct clades (A–D). However, the BI reconstruction had higher values for nodal support than in ML (Fig. 10). In addition, there was a difference within clade A between the 2 phylograms. In the ML reconstruction, *Pseudosanguinicola* was basal to *Sanguinicola* and the subclade containing the present species. In BI, *Pseudosanguinicola* and *Sanguinicola* formed a strongly supported subclade, and were sister to the subclade comprising of the species from the current study. In the 2 reconstructions, the species from the present study clustered with cercariae of ‘*S*. cf. *inermis*’ from Poland (Olson *et al*., 2003) in clade A. The blood fluke sequences from East Africa (‘Sanguinicolid’ sp. W1134 and W1284) formed a distinct and distant clade from well-described Sanguinicolidae spp. Indeed, the 28S rDNA divergence between the East African isolates and sanguinicolids was large (17.2–25.0%). The accession numbers of the sequences and localities of the isolates that were used for phylogenetic analyses are provided in Supplementary file 2.

Partial sequences for CO1 were obtained from 7 isolates of each of the 3 snail species. The representative sequences (641–664 bp) have been submitted to GenBank; accession numbers OR887675-OR887678 (*Bur*. *transvaalensis*), OR887671-OR887674 (*Bur*. *mooiensis*), and OR887679-OR887680 and OR892781-OR892782 (*Bur*. *trapezoidea*). The sequences generated from the current study represent the first published genetic data for the 3 species. Descriptions of the snails are provided (Supplementary file 1). A comprehensive taxonomic and phylogenetic study of the snails will be the subject of a separate study.

## Discussion

### Taxonomy

Considering the paucity of data on genetic characterization of sanguinicolids, the present study is a valuable contribution to the taxonomic study of the group. Data on genetic divergence and the results from phylogenetic analyses showed that the present isolates and ‘*S*. cf. *inermis*’ belong to a genus that is monophyletic with *Sanguinicola* and *Pseudosanguinicola*. Cercaria of Trematoda sp. alias ‘*Sanguinicola*’ sp. from the USA (Zemmer *et al*., 2020) formed a strongly supported subclade with *P*. *occidentalis*, indicating that it is a species of *Pseudosanguinicola*. Sanguinicolidae sp. from Bass, *Micropterus* sp. from the USA (Warren and Bullard, 2023) did not cluster with other isolates from North America, suggesting that it is representative of a distinct genus. The isolates from Vietnam formed a clade with *N. canthoensis*, suggesting that they belong to *Nomasanguinicola*. Freshwater blood flukes from Australia formed 2 separate clades. The first group consists of cercariae of ‘sanguinicolid’ sp. W5003 (Brant *et al*., 2006) and ‘Aporocotylidae’ sp. B, C, D, F and G (Cutmore *et al*., 2023), which seem to represent a single genus within Sanguinicolidae. The second clade consisted of ‘Sanguinicolid’ sp. W5004, which was representative of a taxon that appears to be the most recent ancestor of sanguinicolids. It seems that ‘Sanguinicolid’ sp. W5004 belongs to an unidentified family. Its cercarial features deviate considerably from sanguinicolids. The cercaria was characterized by the presence of prominent eyespots, absence of finfolds on the body and furcae, and a large membrane on either side of the tail stem (Brant *et al*., 2006). These features are absent in cercariae of Sanguinicolidae spp. (Erickson and Wallace, 1959; Schell, 1974; Simon-Martin *et al*., 1987; Kirk and Lewis, 1993; Sendersky and Dobrovolsky, 2004; Faltýnková *et al*., 2007). ‘Sanguinicolid’ sp. W1134 and W1284 from East Africa (Brant *et al*., 2006) formed a distinct clade from all sanguinicolids and appear to be representative of an unknown taxon within an old lineage of freshwater fish blood flukes that is closest to Acipensericolidae. Regarding their morphology, Brant *et al*. (2006) noted the absence of finfolds on the body and furcae of ‘sanguinicolid’ sp. W1284. In contrast, the presence of finfolds on the body and furcae is a common sanguinicolid feature.

The present study is the first to provide genetic data for Sanguinicolidae in Africa. Overall, there are only 3 reports of adult sanguinicolids from Africa, all of which lack genetic characterization. *Sanguinicola chalmersi* Odhner, 1924 was reported in *Auchenoglanis occidentalis* (Valenciennes, 1840) and *Synodontis schall* (Bloch and Schneider, 1801) from Egypt and Sudan (Odhner, 1924; Imam *et al*., 1984; Paperna, 1996). In a recent revision of the genus *Sanguinicola*, Warren *et al*. (2023) noted that *S*. *chalmersi* required more study. Imam *et al*. (1984) described *Sanguinicola clarias* from *Clarias gariepinus* (Burchell, 1822) in Egypt. However, Truong and Bullard (2013) noted that the morphological features of *S*. *clarias* were identical with those of the genus *Nomasanguinicola*. Therefore, its placement in the genus *Sanguinicola* is suspect. Ogambo-Ongoma (1975) reported on the occurrence of an unidentified *Sanguinicola* sp. in tilapiine cichlids from Lake Victoria, East Africa. The morphological descriptions of the sanguinicolid were not provided. Therefore, it appears that at least for now, *S*. *chalmersi* is the only valid representative of the genus *Sanguinicola* in Africa. Based on the present findings, it seems that there are 3 sanguinicolid genera in Africa: *Sanguinicola*, *Nomasanguinicola* and the genus comprising of the species in the present study. Future studies are likely to reveal more genera of blood flukes from African fishes.

Morphological characterization of intramolluscan stages of Digenea often relies on the examination of parthenitae and cercariae using light microscopy (Frandsen and Christensen, 1984; Faltýnkova *et al*., 2007). In the present study, morphological distinctions of the cercariae were made optically, based on overall body size, relative size of the finfolds on the body and furcae, and number of penetration glands in the body. The patterns of papillae and tegumental spines which were clearly discernible only after using SEM, also proved to be distinctive features for species delimitation. Prior to the present study, SEM data were available for cercariae of an unidentified *Sanguinicola* sp. (Simon-Martin and Gomez-Bautista, 1986) and *Sanguinicola inermis* (Kirk and Lewis, 1993). The present study provides additional tegumental features that were not mentioned in previous studies of sanguinicolids. Firstly, the occurrence of 2 groups of transverse circlets on the cephalic organ that differ not only in the size of their spines, but also in number and spacing. Second, the presence of sensilla near the furcal tips and the position of the excretory pore on the furcae.

### Intermediate hosts of sanguinicolids

A vast majority of studies on sanguinicolid larval stages are from Europe, USA and Australia, with minimal reports from Asia, South America and Africa. The intermediate hosts of sanguinicolids from Europe have been presented in detail by Zhokhov *et al*. (2021). The data show that snails from 7 different families (Bithyniidae, Lithoglyphidae, Lymnaeidae, Melanopsidae, Neritidae, Planorbidae, Valvatidae) are intermediate hosts for sanguinicolids (Zhokhov *et al*., 2021). In North America, sanguinicolids have been reported from 4 gastropod families. According to Erickson and Wallace (1959) both *S*. *cristafer* and *S*. *lophophorus* use snails of the genus *Valvata* (Valvatidae) as the intermediate hosts. For *S*. *idahoensis*, the lithoglyphid *F*. *virens* was established to be the intermediate host (Schell, 1974). Preston *et al*. (2021) reported the occurrence of unidentified sanguinicolid cercaria in *Juga plicifera* (Lea, 1838) (Semisulcospiridae) from Oregon, USA. According to Zemmer *et al*. (2020), *Elimia proxima* (Say, 1825) (Pleuroceridae) is the intermediate host for an unidentified sanguinicolid species in Virginia, USA. Studies from Australia show that at least 6 sanguinicolid species use *Posticobia brazieri* (Smith, 1882) (Tateidae) and thiarid *Plotiopsis balonnensis* (Conrad, 1850) as intermediate hosts (Brant *et al*., 2006; Cutmore *et al*., 2023). Reports of intramolluscan stages of freshwater fish blood flukes from South America are very scarce. According to Alda and Martorelli (2014), larvae of an unidentified fluke that was suspected to parasitize freshwater fish were found from *Heleobia australis* (d'Orbigny, 1835) (Cochliopidae) in Argentina. From Asia, larvae of *S*. *lungensis* have been reported only from the lymnaied *Radix plicatula* (Benson, 1842) (Tang and Ling, 1975).

In Africa, reports on sanguinicolids are only from the northeastern and southern parts of the continent. From East Africa, Ogambo-Ongoma (1975) reported *Sanguinicola* larvae in *Radix natalensis* (Krauss, 1848). However, since descriptions of those larvae have not been provided, their species identities are unknown. Morphological data show that *C. sewelli* from *Bur*. *capensis* and *Bur*. *trapezoidea*, *C. britspennata* from *M*. *tuberculata* and *C. capensis* from *Bur*. *Capensis*, which were reported from South Africa (Faust, 1926; Porter, 1938), might be species of Sanguinicolidae. Since sanguinicolids have scarcely been reported in other snail taxa from southern Africa, it seems that *Burnupia* spp. are the main transmitters of sanguinicolids in the region. Although the actual diversity of blood flukes of fish remains unknown in Africa, the group appears to be richer than initially thought. The definitive hosts of the African sanguinicolids are largely unknown. Indeed, the adults of only 2 species have been described from clariid, claroteid and silurid fishes, and only in Egypt and Sudan (Imam *et al*., 1984; Paperna, 1996). Considering the presence of clariid and claroteid fishes in South Africa (Skelton, 2001), we suspect that at least a species of those taxa to be the definitive hosts for the sanguinicolids recorded in the present study.

In summary, sanguinicolids use diverse species belonging to at least 13 different families of pulmonate and caenogastropod snails, as intermediate hosts. To our knowledge, there are no records of sanguinicolids from other invertebrate taxa. Also, it seems that the European sanguinicolids occur in a higher diversity of snail taxa, compared with the other regions of the world. However, this is probably a reflection of the paucity of studies on the intramolluscan stages of freshwater fish blood flukes in the other regions of the world.

## Supporting information

Outa and Avenant-Oldewage supplementary material 1 Outa and Avenant-Oldewage supplementary material

Outa and Avenant-Oldewage supplementary material 2 Outa and Avenant-Oldewage supplementary material

## Data Availability

DNA sequences generated in the present study have been submitted to GenBank: accession numbers OR887671-OR887680, OR892781-OR892782, OR892787-OR892805, OR896075-OR896078 and OR900600.

## Supplementary material

The supplementary material for this article can be found at https://doi.org/10.1017/S003118202300121X

## References

[ref1] Albrecht C, Wilke T, Kuhn K and Streit B (2004) Convergent evolution of shell shape in freshwater limpets: the African genus *Burnupia*. Zoological Journal of the Linnean Society 140, 577–588.

[ref2] Alda P and Martorelli SR (2014) Larval trematodes infecting the South-American intertidal mud snail *Heleobia australis* (Rissooidea: Cochliopidae). Acta Parasitologica 59, 50–67. 24570050 10.2478/s11686-014-0209-3

[ref3] Bouchet P, Rocroi J-P, Hausdorf B, Kaim A, Kano Y, Nützel A, Parkhaev P, Schrödl M and Strong EE (2017) Revised classification, nomenclator and typification of gastropod and monoplacophoran families. Malacologia 61, 1–526.

[ref4] Bouckaert R, Heled J, Kühnert D, Vaughan T, Wu C-H, Xie D, Suchard MA, Rambaut A and Drummond AJ (2014) BEAST 2: a software platform for Bayesian evolutionary analysis. PLoS Computational Biology 10, e1003537. 24722319 10.1371/journal.pcbi.1003537 PMC3985171

[ref5] Brant SV, Morgan JAT, Mkoji GM, Snyder SD, Jayanthe Rajapakse RPV and Loker ES (2006) An approach to revealing blood fluke life cycles, taxonomy, and diversity: provision of key reference data including DNA sequence from single life cycle stages. The Journal of Parasitology 92, 77–88. 16629320 10.1645/GE-3515.1 PMC2519025

[ref6] Brown D (1994) Freshwater Snails of Africa and Their Medical Importance, 2nd Edn. London, UK: Taylor & Francis Ltd.

[ref7] Bullard SA and Overstreet RM (2008) Digeneans as enemies of fishes. In Eiras J, Segner H, Wahil T and Kapoor BG (eds), Fish Diseases. New Hampshire, USA: Science Publishers, pp. 819–974.

[ref8] Connolly M (1939) A monographic survey of South African non-marine molluscs. Annals of the South African Museum 33, 1–660.

[ref9] Craven AE (1881) On a collection of land and freshwater shells from Transvaal & Orange Free State in South Africa, with description of nine new species. Proceedings of Zoological Society of London 1880, 614–618.

[ref10] Cribb TH, Anderson GR, Adlard RD and Bray RA (1998) A DNA-based demonstration of a three-host lifecycle for the Bivesiculidae (Platyhelminthes: Digenea). International Journal for Parasitology 28, 1791–1795. 9846617 10.1016/s0020-7519(98)00127-1

[ref11] Cutmore SC, Littlewood DTJ, Arellano-Martínez M, Louvard C and Cribb TH (2023) Evidence that a lineage of teleost-infecting blood flukes (Aporocotylidae) infects bivalves as intermediate hosts. International Journal for Parasitology 53, 13–25. 36328150 10.1016/j.ijpara.2022.09.007

[ref12] De Kock KN and Wolmarans CT (2009) Distribution of *Burnupia capensis* (Walker, 1912) and *Burnupia stenochorias* (Melvill & Ponsonby, 1903) (Gastropoda: Ancylidae) in South Africa. Suid-Afrikaanse Tydskrif vir Natuurwetenskap en Tegnologie 28, 220–236.

[ref13] Erickson DG and Wallace FG (1959) Studies on blood flukes of the genus *Sanguinicola*. The Journal of Parasitology 45, 310–322. 13665471

[ref14] Faltýnková A, Niewiadomska K, Santos MJ and Valtonen ET (2007) Furcocercous cercariae (Trematoda) from freshwater snails in Central Finland. Acta Parasitologica 52, 310–317.

[ref15] Faust EC (1926) Further observations on South African larval Trematodes. Parasitology 18, 101–127.

[ref16] Folmer O, Black M, Heah W, Lutz R and Vrijenhoek R (1994) DNA primers for amplification of mitochondrial cytochrome C oxidase subunit 1 from diverse metazoan invertebrates. Molecular Marine Biology and Biotechnology 3, 294–299. 7881515

[ref17] Frandsen F and Christensen NO (1984) An introductory guide to the identification of cercariae from African freshwater snails with special reference to cercariae of trematode species of medical and veterinary importance. Acta Tropica 41, 181–202. 6206702

[ref18] Gustinelli A, Caffara M, Florio D, Otachi EO, Wathuta EM and Fioravanti ML (2010) First description of the adult stage of *Clinostomum cutaneum* Paperna, 1964 (Digenea: Clinostomidae) from grey herons *Ardea cinerea* L. and a redescription of the metacercaria from the Nile tilapia *Oreochromis niloticus niloticus* (L.) in Kenya. Systematic Parasitology 76, 39–51. 20401577 10.1007/s11230-010-9231-5

[ref19] Helmer N, Hörweg C, Sattmann H, Reier S, Szucsich NU, Bulantová J and Haring E (2023) DNA barcoding of *Trichobilharzia* (Trematoda: Schistosomatidae) species and their detection in eDNA water samples. Diversity 15, 104.

[ref20] Imam EA, Marzouk MSM, Hassan AA and Itman RH (1984) Studies on *Sanguinicola* sp. (Trematoda) of Nile fishes. Veterinary Medical Journal of Egypt 32, 1–13.

[ref21] Kearse M, Moir R, Wilson A, Stones-Havas S, Cheung M, Sturrock S, Buxton S, Cooper A, Markowitz S, Duran C, Thierer T, Ashton B, Meintjes P and Drummond A (2012) Geneious Basic: an integrated and extendable desktop software platform for the organization and analysis of sequence data. Bioinformatics 28, 1647–1649. 22543367 10.1093/bioinformatics/bts199 PMC3371832

[ref22] Kirk RS and Lewis JW (1993) The life-cycle and morphology of *Sanguinicola inermis* Plehn, 1905 (Digenea: Sanguinicolidae). Systematic Parasitology 25, 125–133. 10.1017/s0031182000060868 1614729

[ref23] Králová-Hromadová I, Špakulová M, Horáčková E, Turčeková Ľ, Novobilsky´ A, Beck R, Koudela B, Marinculic A, Rajsky´ D and Pybus M (2008) Sequence analysis of ribosomal and mitochondrial genes of the giant liverfluke *Fascioloides magna* (Trematoda: Fasciolidae): intraspecific variation and differentiation from *Fasciola hepatica*. Journal of Parasitology 94, 58–67. 18372622 10.1645/GE-1324.1

[ref24] Littlewood DTJ and Olson PD (2001) Small subunit rDNA and the phylum Platyhelminthes: signal, noise, conflict and compromise. In Littlewood DTJ and Bray RA (eds), Interrelationships of the Platyhelminthes. London, UK: Taylor & Francis, pp. 262–278.

[ref25] MolluscaBase (eds) (2021) MolluscaBase. Burnupia Walker, 1912. Available at http://www.molluscabase.org/aphia.php?p=taxdetails&id=933171. Accessed 13 July 2023.

[ref26] Nation JL (1983) A new method using hexamethyldisilazane for preparation of soft insect tissues for scanning electron microscopy. Stain Technology 58, 347–351. 6679126 10.3109/10520298309066811

[ref27] Odhner T (1924) Remarks on *Sanguinicola*. Quarterly Journal of Microscopical Science 68, 403–411.

[ref28] Ogambo-Ongoma AH (1975) Parasitic fish diseases and their impact on potential fish production in East Africa. The African Journal of Tropical Hydrobiology and Fisheries 4, 148–155.

[ref29] Olson PD, Cribb TH, Tkach VV, Bray RA and Littlewood DT (2003) Phylogeny and classification of the Digenea (Platyhelminthes: Trematoda). International Journal of Parasitology 33, 733–755. 12814653 10.1016/s0020-7519(03)00049-3

[ref30] Orélis-Ribeiro R and Bullard SA (2015) Blood flukes (Digenea: Aporocotylidae) infecting body cavity of South American catfishes (Siluriformes: Pimelodidae): two new species from rivers in Bolivia, Guyana and Peru with a re-assessment of *Plehniella* Szidat, 1951. Folia Parasitologica 62, 1–17. 10.14411/fp.2015.050 26373332

[ref31] Orélis-Ribeiro R, Arias CR, Halanych KM, Cribb TH and Bullard SA (2014) Diversity and ancestry of flatworms infecting blood of nontetrapod craniates ‘fishes’. Advances in Parasitology 85, 1–64. 24928179 10.1016/B978-0-12-800182-0.00001-5

[ref32] Outa JO and Avenant-Oldewage A (2023) Integrated characterisation of *Daubaylia burnupiae* n. sp. (Nematoda: Daubayliidae) from a freshwater gastropod in South Africa, with comments on the biology of *Daubaylia* spp. International Journal for Parasitology: Parasites and Wildlife 20, 96–107. 36714046 10.1016/j.ijppaw.2023.01.005 PMC9873581

[ref33] Outa JO, Sattmann H, Köhsler M, Walochnik J and Jirsa F (2020) Diversity of digenean trematode larvae in snails from Lake Victoria, Kenya: first reports and bioindicative aspects. Acta Tropica 206, 105437. 32151590 10.1016/j.actatropica.2020.105437

[ref34] Paperna I (1996) Parasites, infections and diseases of fishes in Africa. An update. Committee for inland fisheries of Africa (CIFA) Technical Paper No. 31, FAO, Rome, 220 pp.

[ref35] Porter A (1938) The larval Trematoda found in certain South African Mollusca with special reference to schistosomiasis (Bilharziasis). South African Institute for Medical Research 42, 1–492.

[ref36] Preston DL, Layden TJ, Segui LM, Falke LP, Brant SV and Novak M (2021) Trematode parasites exceed aquatic insect biomass in Oregon stream food webs. Journal of Animal Ecology 90, 766–775. 33368227 10.1111/1365-2656.13409

[ref37] Schell SC (1974) The life history of *Sanguinicola idahoensis* sp. n. (Trematoda: Sanguinicolidae), a blood parasite of steelhead trout, *Salmo gairdneri* Richardson. The Journal of Parasitology 60, 561–566. 4854934

[ref38] Sendersky IV and Dobrovolsky AA (2004) Morphology and chaetotaxy of *Sanguinicola armata* cercaria (Trematoda: Sanguinicolidae). Parazitologiia 38, 310–321. 15493283

[ref39] Simon-Martin F and Gomez-Bautista M (1986) Preliminary scanning electron microscope observations of the cercaria of a *Sanguinicola* sp. (Digenea: Sanguinicolidae). Annales de Parasitologie Humaine et Comparde 61, 529–531. 10.1051/parasite/1986615529 3813432

[ref40] Simon-Martin F, Rojo-Vazquez FA and Simon-Vicente F (1987) *Sanguinicola rutili* n. sp. (Digenea: Sanguinicolidae) parasito del sistema curculatorio de *Rutilus arcasi* (Cyprinidae) en la provincia de Salamanca. Revista Ibérica de Parasitología 47, 253–261.

[ref41] Skelton PH (2001) A Complete Guide to the Freshwater Fishes of Southern Africa, 2nd Edn. Cape Town, South Africa: Struik Publishers.

[ref42] Tamura T, Stecher G, Peterson D, Filipski A and Kumar S (2013) MEGA 7: molecular evolutionary genetics analysis version 6.0. Molecular Biology and Evolution 30, 2725–2729. 24132122 10.1093/molbev/mst197 PMC3840312

[ref43] Tang CC and Ling SM (1975) *Sanguinicola lungensis* sp. nov. and the outbreaks of sanguinicolosis in Lien-yüe Nursery Ponds in South Fukien. In Chinese, English Abstract. Xiamen Daxne Xuebao 2, 139–160.

[ref44] Tkach VV, Littlewood DTJ, Olson PD, Kinsella JM and Swiderski Z (2003) Molecular phylogenetic analysis of the Microphalloidea Ward, 1901 (Trematoda: Digenea). Systematic Parasitology 56, 1–15. 12975618 10.1023/a:1025546001611

[ref45] Truong TN and Bullard SA (2013) Blood flukes (Digenea: Aporocotylidae) of walking catfishes (Siluriformes: Clariidae): new genus and species from the Mekong River (Vietnam) with comments on related catfish aporocotylids. Folia Parasitologica 60, 237–247. 23951931 10.14411/fp.2013.027

[ref46] Walker B (1912) A revision of the Ancyli of South Africa. Nautilus, Philadelphia 24, 139–144.

[ref47] Warren MB and Bullard SA (2019) First elucidation of a blood fluke (*Electrovermis zappum* n. gen., n. sp.) life cycle including a chondrichthyan or bivalve. International Journal for Parasitology: Parasites and Wildlife 10, 170–183. 31667079 10.1016/j.ijppaw.2019.06.008 PMC6812027

[ref48] Warren MB and Bullard SA (2023) Systematic revision of the fish blood flukes with diagnoses of Chimaerohemecidae Yamaguti, 1971, Acipensericolidae n. fam., Sanguinicolidae poche, 1926, Elopicolidae n. fam., and Aporocotylidae Odhner, 1912. Journal of Parasitology 109, 401–418. 37580059 10.1645/23-13 PMC10658869

[ref49] Warren MB, Poddubnaya LG, Zhokhov AE, Reyda FB, Choudhury A and Bullard SA (2023) Revision of *Sanguinicola* Plehn, 1905 with redescription of *Sanguinicola volgensis* (Rašı´n, 1929) McIntosh, 1934, description of a new species, proposal of a new genus, and phylogenetic analysis. Journal of Parasitology 109, 296–321. 37527276 10.1645/23-14 PMC10658880

[ref50] Zemmer SA, Detwiler JT, Sokol ER, Da Silva Neto JG, Wyderko J, Potts K, Gajewski ZJ, Sarment LV, Benfield EF and Belden LK (2020) Spatial scale and structure of complex lifecycle trematode parasite communities in streams. PLoS ONE 15, e0241973. 33232346 10.1371/journal.pone.0241973 PMC7685432

[ref51] Zhokhov AE, Pugacheva MN and Poddubnaya LG (2021) Freshwater trematodes *Sanguinicola* (Digenea: Aporocotylidae) in Europe: distribution, host range, and characteristics of fish and snail infestation (review). Inland Water Biology 14, 301–315.

