# Supplementary material for: Underreported and taxonomically problematic: characterization of sanguinicolid larvae from freshwater limpets (Burnupiidae), with comments on the phylogeny and intermediate hosts of sanguinicolids
Source: Parasitology. 2023 Nov 29;151(1):108–24. doi: 10.1017/S003118202300121X (PMC10941044; doi:10.1017/S003118202300121X)
Supplement: Outa and Avenant-Oldewage supplementary material 2 — Outa and Avenant-Oldewage supplementary material [file S003118202300121Xsup002.doc]

**Descriptions of *Burnupia* spp. from the present study**

***Burnupia transvaalensis* (Craven, 1880)**

Shell is light brown, ovate, slightly broader on the posterior end. The largest specimen measures 7.0 x 4.5 x 3.2 mm. The apex is about a third the length from the rear margin, subacute and slightly turned towards the right. The lateral slopes are almost equally convex, but the left slope appears more convex than on the right. The anterior slope is convex while the posterior slope is slightly concave and nearly straight in some specimens.


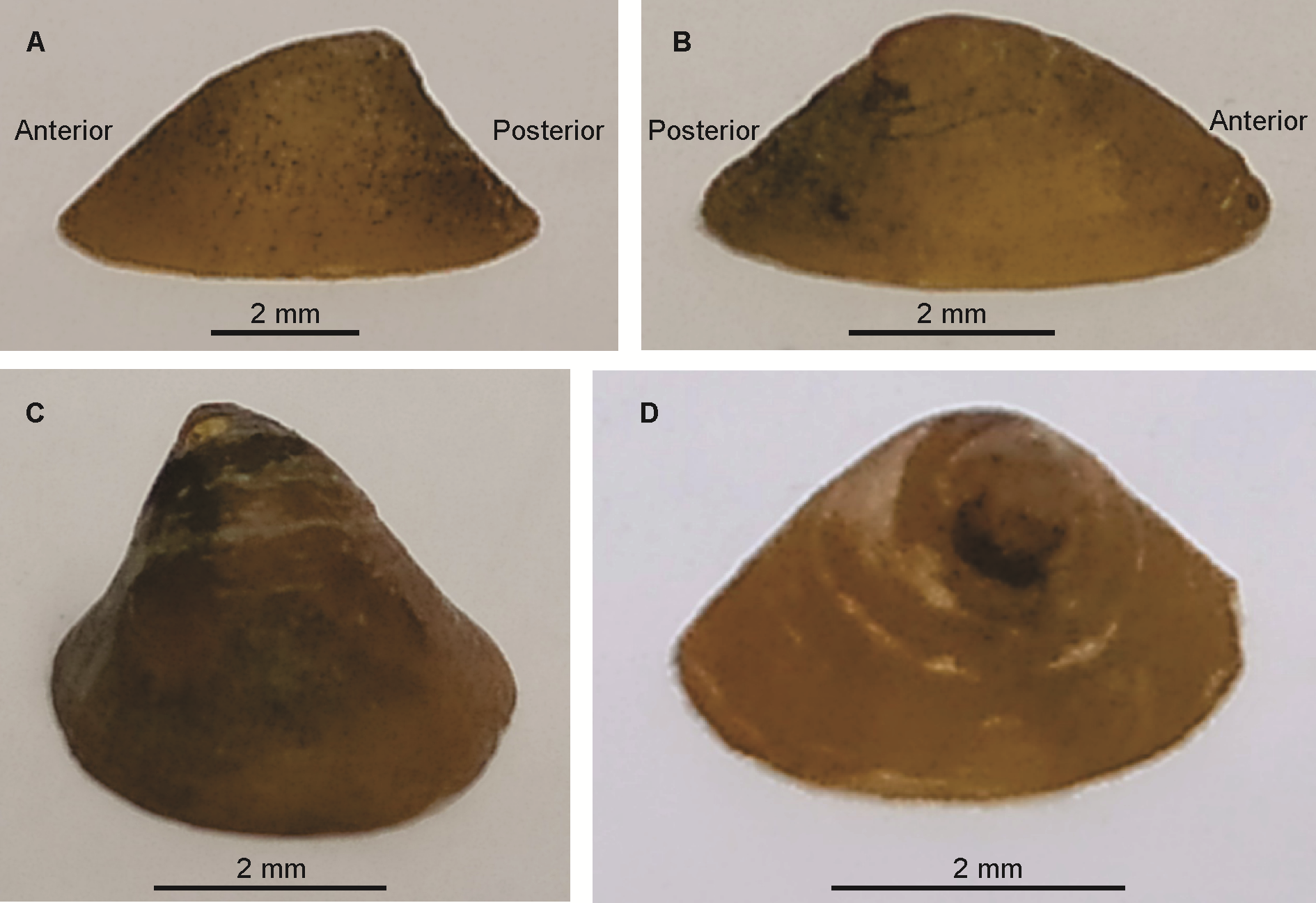


Figure 1. *Burnupia transvaalensis*: A, left side; B, right side; C, anterior end and D, posterior end.

***Burnupia mooiensis* (Walker, 1912)**

Shell, ovate and elevated; largest specimen measures 7.0 x 5.0 x 3.1 mm. The apex is approximately one third the length, from the rear margin; obtuse, weakly beaked and slightly turned to the right. The lateral sides are nearly rounded; however, the left and the anterior slopes are slightly more convex, than the right and the posterior slopes. The posterior slope is slightly concave.


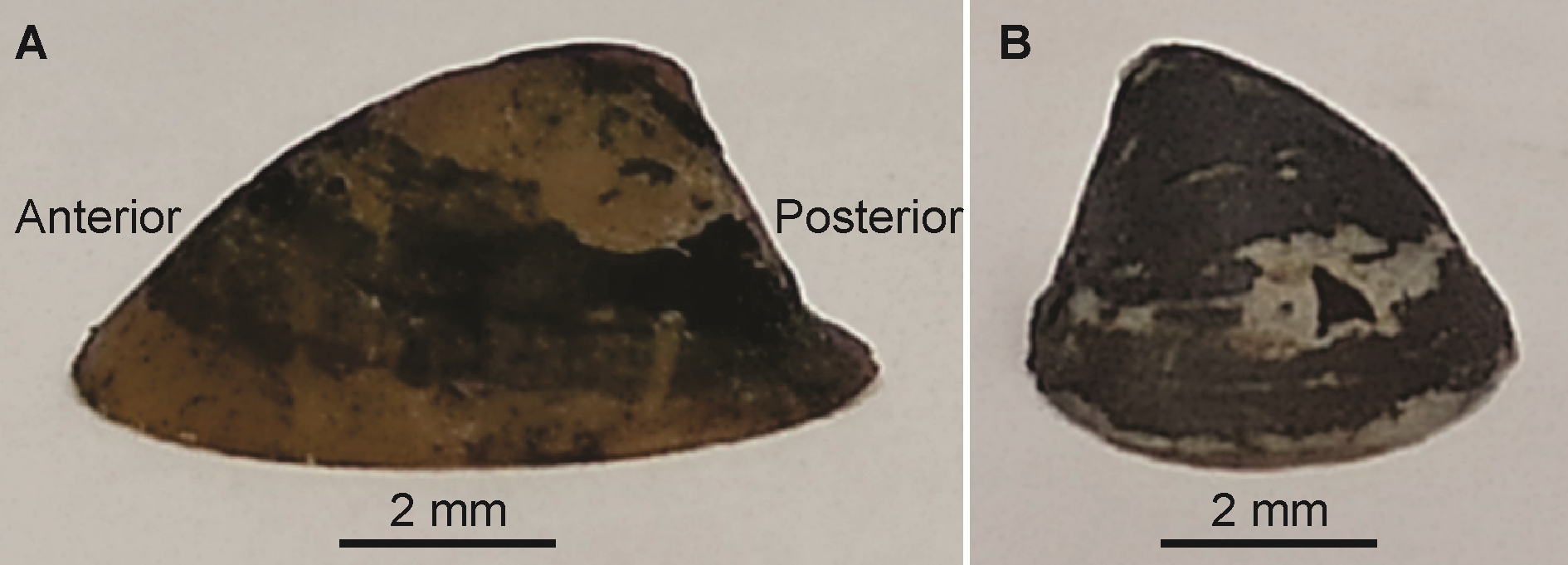


Figure 2. *Burnupia mooiensis*: A, left side and B, anterior end.

***Burnupia* *trapezoidea* (Boettger, 1910)**

Shell, oval and narrow; largest specimen measures 7.5 x 4.3 x 3.5 mm. The apex is closer to the to the posterior end than to the anterior; moderately acute, beaked, and strongly turned to the right. The anterior slope is convex and slightly flattened towards the apex while the posterior slope is concave. The left slope is convex, while the right slope is concave.


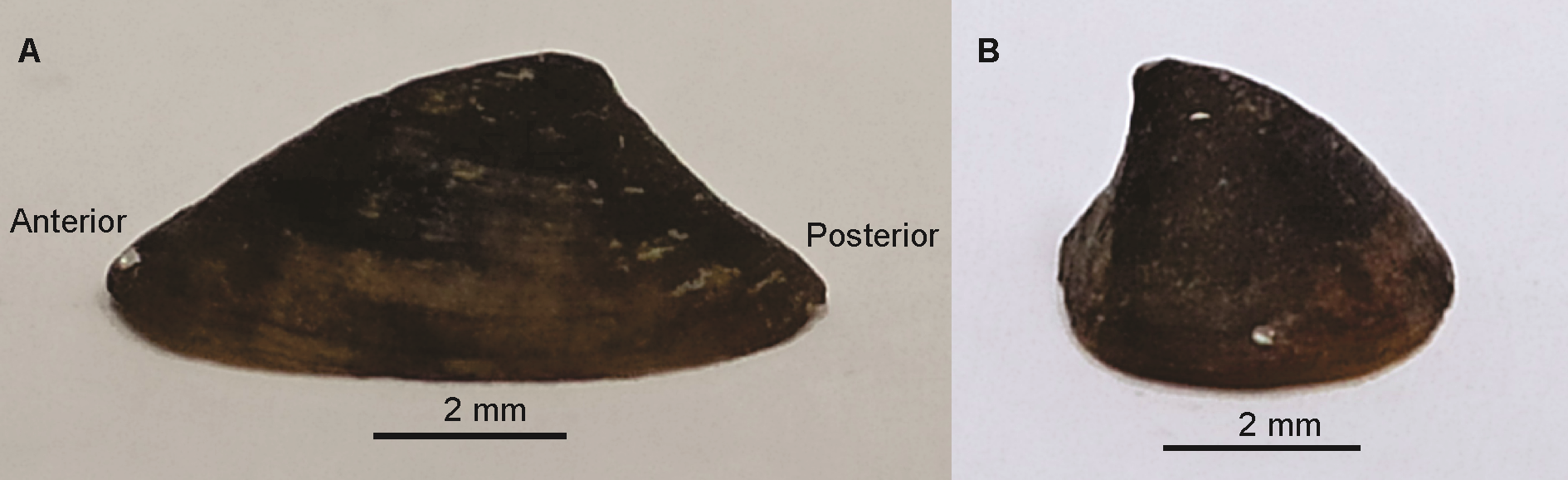


Figure 3. *Burnupia trapezoidea*: A, left side and B, anterior end.
